# Deep homography estimation in dynamic surgical scenes for laparoscopic camera motion extraction

**DOI:** 10.1080/21681163.2021.2002195

**Published:** 2022-02-23

**Authors:** Martin Huber, Sébastien Ourselin, Christos Bergeles, Tom Vercauteren

**Affiliations:** School of Biomedical Engineering & Image Sciences, Faculty of Life Sciences & Medicine, King’s College London, London, UK

**Keywords:** Deep learning, homography estimation, laparoscopic surgery, image processing and analysis, visual data mining and knowledge discovery, virtual reality

## Abstract

Current laparoscopic camera motion automation relies on rule-based approaches or only focuses on surgical tools. Imitation Learning (IL) methods could alleviate these shortcomings, but have so far been applied to oversimplified setups. Instead of extracting actions from oversimplified setups, in this work we introduce a method that allows to extract a laparoscope holder’s actions from videos of laparoscopic interventions. We synthetically add camera motion to a newly acquired dataset of camera motion free da Vinci surgery image sequences through a novel *homography generation algorithm*. The synthetic camera motion serves as a supervisory signal for camera motion estimation that is invariant to object and tool motion. We perform an extensive evaluation of state-of-the-art (SOTA) Deep Neural Networks (DNNs) across multiple compute regimes, finding our method transfers from our camera motion free da Vinci surgery dataset to videos of laparoscopic interventions, outperforming classical homography estimation approaches in both, precision by 41%, and runtime on a CPU by 43%.

## 1. Introduction

The goal in IL is to learn an expert policy from a set of expert demonstrations. IL has been slow to transition to interventional imaging. In particular, the slow transition of modern IL methods into automating laparoscopic camera motion is due to a lack state-action-pair data (Kassahun et al. 2016; Esteva et al. 2019). The need for automated laparoscopic camera motion (Pandya et al. 2014; Ellis et al. 2016) has, therefore, historically sparked research in rule-based approaches that aim to reactively centre surgical tools in the field of view (Agustinos et al. 2014; Da Col et al. 2020). DNNs could contribute to this work by facilitating SOTA tool segmentations and automated tool tracking (Garcia-Peraza-Herrera et al. 2017, 2021; Gruijthuijsen et al. 2021).

Recent research contextualises laparoscopic camera motion with respect to (w.r.t.) the user and the state of the surgery. DNNs could facilitate contextualisation, as indicated by research in surgical phase and skill recognition (Kitaguchi et al.^2020^). However, current contextualisation is achieved through handcrafted rule-based approaches (Rivas-Blanco et al. 2014, 2017), or through stochastic modelling of camera positioning w.r.t. the tools (Weede et al. 2011; Rivas-Blanco et al. 2019). While the former do not scale well and are prone to nonlinear interventions, the latter only consider surgical tools. However, clinical evidence suggests camera motion is also caused by the surgeon’s desire to observe tissue (Ellis et al. 2016). Non-rule-based, i.e. IL, attempts that consider both, tissue, and tools as source for camera motion are Ji et al. 2018; Su et al. 2020; Wagner et al. 2021, but they utilise an oversimplified setup, require multiple cameras or tedious annotations.

In current laparoscopic camera motion automation, DNNs merely solve auxiliary tasks. Consequentially, current laparoscopic camera motion automation is rule-based, and disregards tissue. While modern IL approaches could alleviate these issues, clinical data of laparoscopic surgeries remains unusable for IL. Therefore, SOTA IL attempts rely on artificially acquired data (Ji et al. 2018; Su et al. 2020; Wagner et al. 2021).

In this work, we aim to extract camera motion from videos of laparoscopic interventions, thereby creating state-action-pairs for IL. To this end, we introduce a method that isolates camera motion (actions) from object and tool motion by solely relying on observed images (states). To this end, DNNs are supervisedly trained to estimate camera motion while disregarding object, and tool motion. This is achieved by synthetically adding camera motion via a novel *homography generation algorithm* to a newly acquired dataset of camera motion free da Vinci surgery image sequences. In this way, object, and tool motion reside within the image sequences, and the synthetically added camera motion can be regarded as the only source, and therefore ground truth, for camera motion estimation. Extensive experiments are carried out to identify modern network architectures that perform best at camera motion estimation. The DNNs that are trained in this manner are found to generalise well across domains, in that they transfer to vast laparoscopic datasets. They are further found to outperform classical camera motion estimators.

## 2. Related work

Supervised deep homography estimation was first introduced in (DeTone et al. 2016) and got improved through a hierarchical homography estimation in (Erlik Nowruzi et al. 2017). It got adopted in the medical field in (Bano et al. 2020). All three approaches generate a limited set of homographies, only train on static images, and use non-SOTA VGG-based network architectures (Simonyan and Zisserman 2014).

Unsupervised deep homography estimation has the advantage to be applicable to unlabelled data, e.g. videos. It was first introduced in (Nguyen et al. 2018), and got applied to endoscopy in (Gomes et al. 2019). The loss in image space, however, can’t account for object motion, and only static scenes are considered in their works. Consequentially, recent work seeks to isolate object motion from camera motion through unsupervised incentives. Closest to our work are Le et al. (2020), where the authors generate a dataset of camera motion free image sequences. However, due to tool, and object motion, their data generation method is not applicable to laparoscopic videos, since it relies on motion free image borders. Zhang et al. (2020) provide the first work that does not need a synthetically generated dataset. Their method works fully unsupervised, but constraining what the network minimises, is difficult to achieve.

Only (Le et al. 2020) and (Zhang et al. 2020) train DNNs on object motion invariant homography estimation. Contrary to their works, we train DNNs supervisedly. We do so by applying the data generation of DeTone et al. (2016) to image sequences rather than single images. We further improve their method by introducing a novel *homography generation algorithm* that allows to continuously generate synthetic homographies at runtime, and by using SOTA DNNs.

## 3. Materials and methods

### 3.1. Theoretical background

Two images are related by a homography if both images view the same plane from different angles and distances. Points on the plane, as observed by the camera from different angles in homogeneous coordinates $p_{i}={\left[\begin{array}{ccc} u_{i} & v_{i} & 1 \end{array}\right]}^{T}$ are related by a projective homography G (Malis and Vargas 2007)

(1) $$\alpha_{g}p_{i}={{Gp}_{i}}^{\prime }.$$

Since the points $p_{i}$ and ${p_{i}}^{\prime }$ are only observed in the 2D image, depth information is lost, and the projective homography G can only be determined up to scale $\alpha_{g}$. The distinction between projective homography G and homography in Euclidean coordinates $H=K^{-1}GK$, with the camera intrinsics K, is often not made for simplicity, but is nonetheless important for control purposes Huber, et al., 2021. The eight unknown parameters of G can be obtained through a set of N≥4 matching points P = $\left\{(p_{i},{p_{i}}^{\prime }),i\in \;\left[0,N-1\right]\right\}$ by rearranging (1) into

(2) $$\left[\begin{array}{ccccccccc} {u_{i}}^{\prime } & {v_{i}}^{\prime } & 1 & 0 & 0 & 0 & -{u_{i}}^{\prime }u_{i} & -{v_{i}}^{\prime }u_{i} & -u_{i}\\ 0 & 0 & 0 & {u_{i}}^{\prime } & {v_{i}}^{\prime } & 1 & -{u_{i}}^{\prime }v_{i} & -{v_{i}}^{\prime }v_{i} & -v_{i} \end{array}\right]g=0\;\forall i,$$

where g holds the entries of G as a column vector. The ninth constraint, by convention, is usually to set $||g|{|}_{2}=1$. Classically, P is obtained through feature detectors but it may also be used as a means to parameterise the spatial transformation. Recent deep approaches indeed set P as the corners of an image, and predict $\Delta p_{i}={p_{i}}^{\prime }-p_{i}$. This is also known as the four point homography $G_{4point}$

(3) $$G_{4point}=\left[\begin{array}{cc} \Delta u_{0} & \Delta v_{0}\\ \Delta u_{1} & \Delta v_{1}\\ \Delta u_{2} & \Delta v_{2}\\ \Delta u_{3} & \Delta v_{3} \end{array}\right],$$

which relates to G through (2), where ${p_{i}}^{\prime }=p_{i}+\Delta p_{i}$.

### 3.2. Data preparation

**Figure 1.**
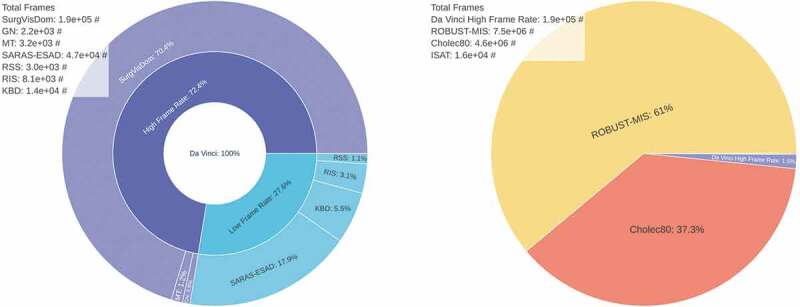
Da Vinci surgery and laparoscopic surgery datasets, referring to Section 3.2. Shown are relative sizes and the absolute number of frames. Da Vinci surgery datasets (left). Included are: SurgVisDom Zia, et al., 2021, GN Giannarou, et al., 2012, MT Mountney, et al., 2010, SARAS-ESAD Bawa, et al., 2020, KBD Hattab, et al., 2020, RIS Allan, et al., 2019, and RSS Allan, et al., 2020. They are often released at a low frame rate of 1fps for segmentation tasks. Much more laparoscopic surgery data is available (right). Included are ROBUST-MIS Maier-Hein, et al., 2020, Cholec80 Twinanda, et al., 2016, ISAT Bodenstedt, et al., 2018, and the HFR da Vinci dataset from (left) for reference.

Similar to (Le et al. 2020), we initially find camera motion free image sequences, and synthetically add camera motion to them. In our work, we isolate camera motion free image sequences from da Vinci surgeries, and learn homography estimation supervisedly. We acquire publicly available laparoscopic, and da Vinci surgery videos. An overview of all datasets is shown in Figure 1. Excluded are synthetic, and publicly unavailable datasets. Da Vinci surgery datasets, and laparoscopic surgery datasets require different pre-processing steps, which are described below.

#### 3.2.1. Da Vinci surgery data pre-processing

Many of the da Vinci surgery datasets are designed for tool or tissue segmentation tasks, therefore, they are published at a frame rate of 1fps, see Figure 1 (left). We merge all high frame rate (HFR) datasets into a single dataset and manually remove image sequences with camera motion, which amount to 5% of all HFR data. We crop the remaining data to remove status indicators, and scale the images to 306×408 pixels, later to be cropped by the *homography generation algorithm* to a resolution of 240×320.

#### 3.2.2. Laparoscopic surgery data pre-processing

**Figure 2.**
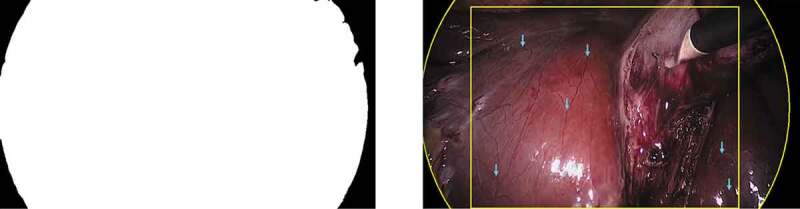
Cholec80 dataset pre-processing, referring to Section 3.2.2. The black boundary circle is automatically detected through fitting a circle to the binary segmentation. The binary segmentation mask is shown on the left. The circular boundary fitting and static landmarks (blue arrows) are shown on the right. Landmarks are manually annotated and tracked over time.

Laparoscopic images are typically observed through a Hopkins telescope, which causes a black circular boundary in the view, see Figure 2. This boundary does not exist in da Vinci surgery recordings. For inference on the laparoscopic surgery image sequences, the most straightforward approach is to crop the view. To this purpose, we determine the centre and radius of the circular boundary, which is only partially visible. We detect it by randomly sampling N points $p_{i}=(u_{i},v_{i}{)}^{T}$ on the boundary. This is similar to work in (Münzer et al. 2013), but instead of computing an analytical solution, we fit a circle by means of a least squares solution through inversion of

(4) $$\left[\begin{array}{ccc} 2u_{0} & 2v_{0} & 1\\ & \vdots & \\ 2u_{N-1} & 2v_{N-1} & 1 \end{array}\right]\left[\begin{array}{c} x_{0}\\ x_{1}\\ x_{2} \end{array}\right]=\left[\begin{array}{c} u_{0}^{2}+v_{0}^{2}\\ \vdots \\ u_{N-1}^{2}+v_{N-1}^{2} \end{array}\right],$$

where the circle’s centre is $\left(x_{0},x_{1}\right)$, and its radius is $\sqrt{x_{2}+x_{0}^{2}+x_{1}^{2}}$. We then crop the view centrally around the circle’s centre, and scale it to a resolution of 240×320. An implementation is provided on GitHub.^1^

#### 3.2.3. Ground truth generation

One can simply use the synthetically generated camera motion as ground truth at train time. For inference on the laparoscopic dataset, this is not possible. We therefore generate ground truth data by randomly sampling 50 image sequences with 10 frames each from the Cholec80 dataset. In these image sequences, we find characteristic landmarks that are neither subject to tool, nor to object motion, see Figure 2 (right). Tracking of these landmarks over time allows one to estimate the camera motion in between consecutive frames through (2).

### 3.3. Deep homography estimation

**Figure 3.**
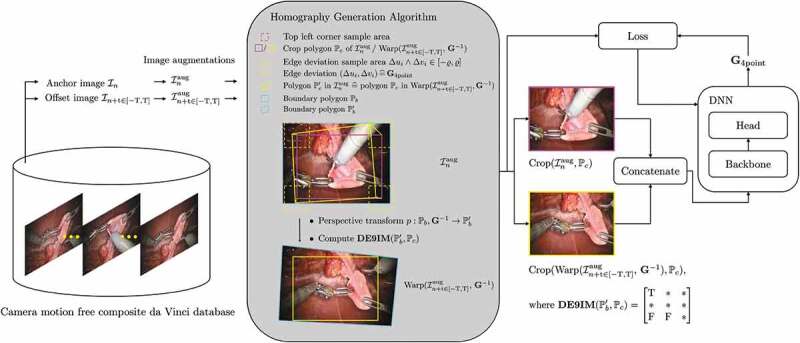
Deep homography estimation training pipeline, referring to Section 3.3. Image pairs are sampled from the HFR da Vinci surgery dataset. The *homography generation algorithm* then adds synthetic camera motion to the augmented images, which is regressed through a backbone DNN.

In this work we exploit the static camera in da Vinci surgeries, which allows us to isolate camera motion free image sequences. The processing pipeline is shown in Figure 3.

Image pairs are sampled from image sequences of the HFR da Vinci surgery dataset of Figure 1. An image pair consists of an anchor image $I_{n}$, and an offset image $I_{n+t}$. The offset image is sampled uniformly from and interval t∈[−T,T] around the anchor. The HFR da Vinci surgery dataset is relatively small, compared to the laparoscopic datasets, see Figure 1 (right). Therefore, we apply image augmentations to the sampled image pairs. They include transform to greyscale, horizontal, and vertical flipping, cropping, change in brightness, and contrast, Gaussian blur, fog simulation, and random combinations of those. Camera motion is then added synthetically to the augmented image $I_{n+t}^{aug}$ via the *homography generation algorithm* from Section 3.4. A DNN, with a backbone, then learns to predict the homography $G_{4point}$ between the augmented image, and the augmented image with synthetic camera motion at time step n+t.

### 3.4. Homography generation algorithm

In its core, the *homography generation algorithm* is based on the works of DeTone et al. 2016. However, where DeTone et al. crop the image with a safety margin, our method allows to sample image crops across the entire image. Additionally, our method computes feasible homographies at runtime. This allows us to continuously generate synthetic camera motion, rather then training on a fixed set of precomputed homographies. The *homography generation algorithm* is summarised in Alg. 1, and visualised in Figure 3.

Initially, a *crop polygon* $P_{c}$ is generated for the augmented image $I_{n}^{aug}$. The *crop polygon* is defined through a set of points in the augmented image $P_{c}=\{p_{i}^{c},\;i\in \left[0,3\right]\}$, which span a rectangle. The top left corner $p_{0}^{c}$ is randomly sampled such that the *crop polygon* $P_{c}$ resides within the image border polygon $P_{b}$, hence $p_{0}^{c}\in ([0,h_{b}-h_{c}],[0,w_{b}-w_{c}])$, where h, and w are the height and width of the *crop*, and the *border polygon*, respectively. Following that, a random four point homography $G_{4point}$ (3) is generated by sampling edge deviations $\Delta u_{i}\wedge \Delta v_{i}\in \left[-\varrho ,\varrho \right]$. The corresponding inverse homography $G^{-1}$ is used to warp each point of the border polygon $P_{b}$ to ${P_{b}}^{\prime }$. Finally, the Dimensionally Extended 9-Intersection Model (Clementini et al. 1994) is used to determine whether the warped polygon ${P_{b}}^{\prime }$ contains $P_{c}$, for which we utilise the Python library *Shapely*.^2^ If the thus found intersection matrix DE9IM satisfies

(5) $$DE9IM({P_{b}}^{\prime },P_{c})=\left[\begin{array}{c} T\\ {}*\\ F \end{array}\begin{array}{c} {}*\\ {}*\\ F \end{array}\begin{array}{c} {}*\\ {}*\\ {}* \end{array}\right]$$

the homography $G^{-1}$ is returned, otherwise a new four point homograpy $G_{4point}$ is sampled. Therein, ∗ indicates that the intersection matrix may hold any value, and T,F indicate that the intersection matrix must be true or false at the respective position. In the unlikely case that no homography is found after *maximum rollouts*, the identity $G_{4point}=0$ is returned. Once a suitable homography is found, a crop of the augmented image $Crop(I_{n}^{aug},P_{c})$ is computed, as well as a crop of the warped augmented image at time n+t, $Crop(Warp(I_{n+t}^{aug},G^{-1}),P_{c})$. This keeps all computationally expensive operations outside the loop.

**Table:** 

**Algorithm 1**: Homography generation algorithm, referring to Section 3.4.
Randomly sample crop polygon $P_{c}$ of desired shape in $P_{b}$;
**while** rollouts < maximum rollouts **do**
Randomly sample $G_{4point}$, where $\Delta u_{i}\wedge \Delta v_{i}\in \left[-\varrho ,\varrho \right]\forall i$;
Perspective transform boundary polygon $p:P_{b},G^{-1}\to {P_{b}}^{\prime }$;
Compute intersection matrix $DE9IM({P_{b}}^{\prime },P_{c})$;
**if $DE9IM=\left[\begin{array}{c} T\\ {}*\\ F \end{array}\begin{array}{c} {}*\\ {}*\\ F \end{array}\begin{array}{c} {}*\\ {}*\\ {}* \end{array}\right]$ then**
**return**$G_{4point}$, $P_{c}$;
**end**
Increment *rollouts*;
**end**
**return**0, $P_{c}$;

## 4. Experiments

We train DNNs on a 80% train split of the HFR da Vinci surgery dataset from Figure 1. The 20% test split is referred to as test set in the following. Inference is performed on the ground truth set from Section 3.2.3. We compute the Mean Pairwise Distance (MPD) of the predicted value for $G_{4point}$ from the desired one. We then compute the Cumulative Distribution Function (CDF) of all MPDs. We evaluate the CDF at different thresholds $t_{i},\;i\in \left\{30,50,70,90\right\}$, e.g. 30% of all homography estimations are below a MPD of $t_{30}$. We additionally evaluate the compute time on a GeForce RTX 2070 GPU, and a Intel Core i7-9750 H CPU.

### 4.1. Backbone search

In this experiment, we aim to find the best performing backbone for homography estimation. Therefore, we run the same experiment repeatedly with fixed hyperparameters, and varying backbones. We train each network for 50 epochs, with a batch size of 64, using the Adam optimiser with a learning rate of 2e−4. The edge deviation ϱ is set to 32, and the sequence length T to 25.

### 4.2. Homography generation algorithm

In this experiment, we evaluate the *homography generation algorithm*. For this experiment we fix the backbone to a ResNet-34, and train it for 100 epochs, with a batch size of 256, using the Adam optimiser with a learning rate of 1e-3. Initially, we fix the sequence length T to 25, and train on different edge deviations $\varrho \in \left\{32,48,64\right\}$. Next, we fix the edge deviation ϱ to 48, and train on different sequence lengths T∈{1,25,50}, where a sequence length of 1 corresponds to a static pair of images.

## 5. Results

### 5.1. Backbone search

The results are listed in Table 1. It can be seen that the deep methods generally outperform the classical methods on the test set. There is a tendency that models with more parameters perform better. On the ground truth set, this tendency vanishes. The differences in performance become independent of the number of parameters. Noticeably, many backbones still outperform the classical methods across all thresholds on the ground truth set, and low compute regime models also run quicker on CPU than comparable classical methods. E.g. we find that EfficientNet-B0, and RegNetY-400MF run at 36Hz, and 50Hz on a CPU, respectively. Both outperform SURF & RANSAC in homography estimation, which runs at 20Hz.

**Table 1.** Results referring to Section 5.1. All methods are tested on the da Vinci HFR test set, indicated by $t_{i}^{test}$, and the Cholec80 inference set, indicated by $t_{i}^{gt}$. Best, and second best metrics are highlighted with bold character. Improvements in precision $t_{90,imp}^{gt}$ and compute time $CPU_{imp}$ are given w.r.t. SURF & RANSAC

Name	$t_{30}^{test}/t_{30}^{gt}\;[pixels]$	$t_{50}^{test}/t_{50}^{gt}\;[pixels]$	$t_{70}^{test}/t_{70}^{gt}\;[pixels]$	$t_{90}^{test}/t_{90}^{gt}\;[pixels]$	$t_{90,imp}^{gt}\;[\%]$	params[M]	flops[M]	GPU[ms]	CPU[ms]	$CPU_{imp}\;[\%]$
VGG-style	4.83/2.45	6.47/2.94	8.68/3.59	13.23/5.41	−60	92.92	11.12	2±1	83±2	−69±33
ResNet-18	1.42/1.12	1.95/1.33	2.82/1.58	5.06/2.20	35	11.19	6.02	3±1	31±3	38±13
ResNet-34	1.33/1.02	1.81/1.19	2.56/1.52	4.63/2.08	39	21.3	11.74	6±1	51±5	−3±23
ResNet-50	1.40/1.08	1.89/1.33	2.70/1.57	4.79/2.21	35	23.53	13.12	10±1	72±4	−46±29
EfficientNet-B0	1.36/1.09	1.83/1.31	2.62/1.50	4.64/2.01	41	4.02	1.28	12±2	28±2	43±12
EfficientNet-B1	1.32/1.02	1.77/1.26	2.50/1.57	4.42/2.01	41	6.52	1.88	17±1	37±1	25±15
EfficientNet-B2	1.40/1.06	1.85/1.29	2.57/1.55	4.42/2.15	37	7.71	2.16	17±2	41±1	18±16
EfficientNet-B3	1.31/1.05	1.75/1.36	2.44/1.68	4.23/2.26	33	10.71	3.14	20±2	55±4	−11±23
EfficientNet-B4	1.23/1.08	1.65/1.31	2.29/1.69	4.02/2.14	37	17.56	4.88	24±2	68±5	−38±29
EfficientNet-B5	1.26/1.18	1.67/1.35	2.30/1.65	4.02/2.06	39	28.36	7.62	29±2	93±5	−89±37
RegNetY-400MF	1.55/1.01	2.07/1.29	2.90/1.60	5.08/2.12	37	3.91	1.32	13±1	20±1	58±8
RegNetY-600MF	1.47/1.03	1.98/1.28	2.80/1.56	4.87/2.21	35	5.45	1.94	13±1	24±3	52±11
RegNetY-800MF	1.43/1.08	1.92/1.32	2.70/1.59	4.76/2.12	37	5.50	2.54	12±1	24±1	51±10
RegNetY-1.6GF	1.38/1.03	1.83/1.27	2.52/1.60	4.35/2.16	36	10.32	5.08	21±2	42±4	16±18
RegNetY-4.0GF	1.27/1.05	1.69/1.26	2.36/1.66	4.17/2.17	36	19.57	12.36	21±2	66±5	−34±28
RegNetY-6.4GF	1.21/1.04	1.64/1.27	2.32/1.60	4.17/2.09	38	29.30	19.72	25±3	98±6	−100±40
SURF & RANSAC	4.06/1.07	5.65/1.40	7.93/2.02	13.62/3.39	0	N/A	N/A	N/A	49±9	0±27
SIFT & RANSAC	4.28/1.25	6.02/1.76	8.65/2.48	16.52/4.63	−37	N/A	N/A	N/A	37±9	25±22
ORB & RANSAC	6.52/1.65	10.48/2.47	20.12/3.71	122.66/6.81	−101	N/A	N/A	N/A	12±2	76±6

### 5.2. Homography generation algorithm

**Figure 4.**
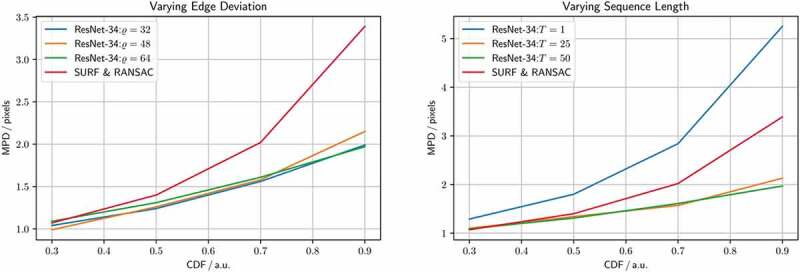
Homography generation optimisation, referring to Section 5.2. Shown is a ResNet-34 homography estimation for different homography generation configurations, and a SURF & RANSAC homography estimation for reference. Varying edge deviation $\varrho \in \left\{32,48,64\right\}$, and fixed sequence length T=25 (left). Varying sequence length $T\in \left\{1,25,50\right\}$, and fixed edge deviation ϱ=48 (right).

**Figure 5.**
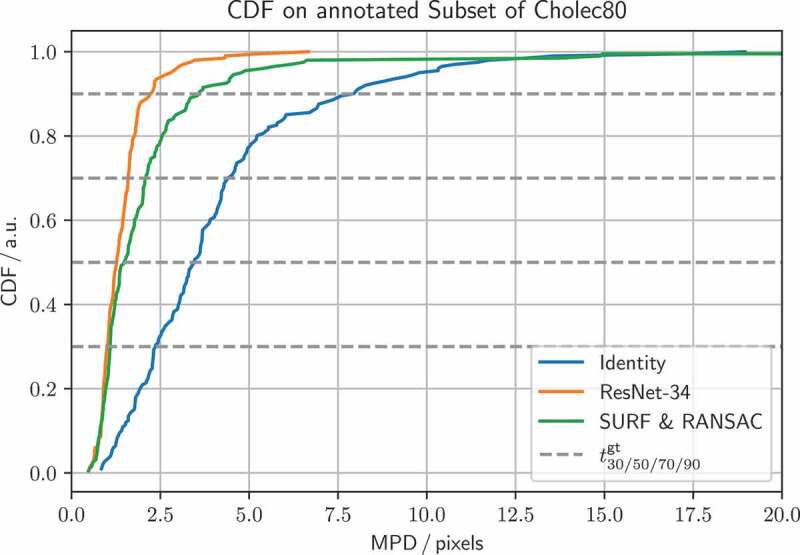
CDF for SURF & RANSAC, and ResNet-34, trained with a sequence length T=25, and edge deviation ϱ=48, referring to Section 5.2. The identity is added for reference. CDF thresholds for the SURF & RANSAC are $t_{1/10/30/50/70/90}^{gt}=0.51/0.80/1.09/1.48/2.07/3.53\;pixels$, and for the ResNet-34 $t_{1/10/30/50/70/90}^{gt}=0.50/0.83/1.00/1.26/1.59/2.15\;pixels$. ResNet-34 generally performs better, and has no outliers.

**Figure 6.**
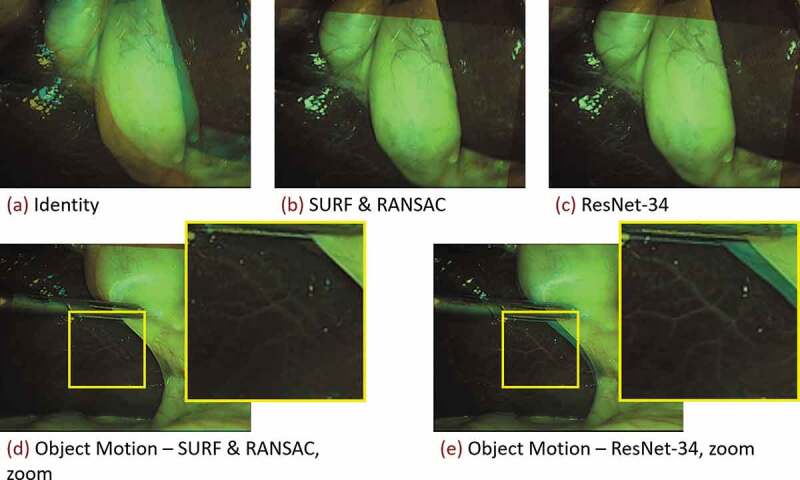
Classical homography estimation using a SURF feature detector under RANSAC outlier rejection, and the proposed deep homography estimation with a ResNet-34 backbone, referring to Section 5.2. Shown are blends of consecutive images from a 5fps resampled Cholec80 exemplary sequence (Twinanda et al. 2016). Decreasing the framerate from originally 25fps to 5fps, increases the motion in between consecutive frames. (Top row) Homography estimation under predominantly camera motion. Both methods perform well. (Bottom row) Homography estimation under predominantly object motion. Especially in the zoomed images it can be seen that the classical method (d) misaligns the stationary parts of the image, whereas the proposed method (e) aligns the background well.

Given that ResNet-34 performs well on the ground truth set, and executes fast on the GPU, we run the *homography generation algorithm* experiments with it. It can be seen in Figure 4 (left), that the edge deviation ϱ is neglectable for inference. In Figure 4 (right), one sees the effects of the sequence length T on the inference performance. Notably, with T=1, corresponding to static image pairs, the SURF & RANSAC homography estimation outperforms the ResNet-34. For the other sequence lengths, ResNet-34 outperforms the classical homography estimation. The CDF for the best performing combination of parameters, with T=25, and ϱ=48, is shown in Figure 5. Our method generally outperforms SURF & RANSAC. The advantage of our method becomes most apparent for a CDF≥0.5. Even the identity outperforms SURF & RANSAC for large MPDs. This aligns with the qualitative observation that motion is often overestimated by SURF & RANSAC, which is shown in Figure 6. An exemplary video is provided.^3^

## 6. Discussion

In this work we supervisedly learn homography estimation in dynamic surgical scenes. We train our method on a newly acquired, synthetically modified da Vinci surgery dataset and successfully cross the domain gap to videos of laparoscopic surgeries. To do so, we introduce extensive data augmentation and continuously generate synthetic camera motion through a novel *homography generation algorithm*.

In Section 5.1, we find that, despite the domain gap for the ground truth set, DNNs outperform classical methods, which is indicated in Table 1. The homography estimation performance proofs to be independent of the number of model parameters, which indicates an overfit to the test data. The independence of the number of parameters allows to optimise the backbone for computational requirements. E.g. a typical laparoscopic setup runs at 25−30Hz, the classical method would thus already introduce a bottleneck at 20Hz. On the other hand, EfficientNet-B0, with 36Hz, and RegNetY-400MF, with 50Hz, introduce no latency, and could be integrated into systems without GPU.

In Section 5.2, we find that increasing the edge deviation has no effect on the homography estimation, see Figure 4 (left). This is because the motion in the ground truth set does not exceed the motion in the training set. In Figure 4 (right), we further find how training DNNs on synthetically modified da Vinci surgery image sequences enables our method to isolate camera from object and tool motion, validating our method. In Figure 5, it is demonstrated that ResNet-34 generally outperforms SURF & RANSAC. This shows that generating camera motion synthetically through homographies, which approximates the surgical scene as a plane, does not pose an issue.

The object, and tool motion invariant camera motion estimation allows one to extract a laparoscope holder’s actions from videos of laparoscopic interventions, which enables the generation of image-action-pairs. In future work, we will generate image-action-pairs from laparoscopic datasets and apply IL to them. Describing camera motion (actions) by means of a homography is grounded in recent research for robotic control of laparoscopes (Huber et al. 2021). This work will therefore support the transition towards robotic automation approaches. It might further improve augmented reality, and image mosaicing methods in dynamic surgical environments.

## Notes

1. https://github.com/RViMLab/endoscopy.

2. https://pypi.org/project/Shapely.

3. https://drive.google.com/file/d/1totjHbhIMEL7a-QAiL7B1rT44wvWB6lO/view?usp=sharing.
